# ﻿Towards a better knowledge and conservation of cryptic macrolichens in Italy: a revision of the genus *Cetrelia* (Parmeliaceae, Lecanorales, lichenized Ascomycota)

**DOI:** 10.3897/mycokeys.120.154233

**Published:** 2025-08-08

**Authors:** Gabriele Gheza, Chiara Vallese, Luca Di Nuzzo, Simona Corneti, Renato Benesperi, Elisabetta Bianchi, Giulia Canali, Silvia Del Vecchio, Luana Francesconi, Paolo Giordani, Pier Luigi Nimis, Walter Obermayer, Chiara Pistocchi, Helmut Mayrhofer, Juri Nascimbene

**Affiliations:** 1 BIOME Lab, Department of Biological, Geological and Environmental Sciences, Alma Mater Studiorum, University of Bologna, Bologna, Italy; 2 Department of Earth, Environment and Life Sciences (DISTAV), University of Genova, Genova, Italy; 3 Department of Biology, University of Firenze, Firenze, Italy; 4 Department of Pharmacy, University of Genova, Genova, Italy; 5 Department of Life Sciences, University of Trieste, Trieste, Italy; 6 Institute of Biology, University of Graz, Graz, Austria

**Keywords:** Alps, Apennines, Lichens, Mediterranean

## Abstract

Cryptic species are a challenge for conservation since their ambiguous recognition can hinder a reliable evaluation of their distribution and ecology, thus affecting the assessment of their conservation status. *Cetrelia* W.L. Culb. & C.F. Culb. is a foliose chlorolichen genus with four species in Europe, which represents a good case-study on this issue. All four sorediate *Cetrelia* species are morphologically very similar and also show a similar ecology. They can be identified by chemical characters related to their distinctive secondary metabolites, whose diagnostic value is also supported by molecular data. In addition, they are overall rare, and therefore virtually endangered, although in previous assessments they were evaluated as “data deficient” due to the scarcity of available data. The few, old literature records in Italy refer almost exclusively to one species (*C.olivetorum*), which, however, has been shown to be quite rare in other European countries. To better elucidate the actual distribution of the four species in Italy, we carried out a revision of all the available herbarium specimens and checked several new collections from the main centres of distribution. We analysed 320 specimens from 59 sites, confirming the occurrence of all the four species reported from Europe. *Cetreliamonachorum* is the most widespread, ranging from the Alps to the Apennines and Sardegna. *Cetreliacetrarioides* is less widespread, occurring across the Italian Alps. *Cetreliaolivetorum* is confined to the Eastern Alps and northern Apennines. *Cetreliachicitae* is the rarest, being found only in five sites in the Central and Eastern Alps. All the four species dwell in old, moist montane forests dominated by beech and/or conifers and with long ecological continuity, but they show different biogeographical patterns, which should be considered for planning conservation actions. All the sites hosting *Cetrelia* species, especially those in which more than one species occur, would deserve protection.

## ﻿Introduction

Cryptic species represent a challenge for conservation, since their difficult recognition can hinder a reliable evaluation of their distribution and ecology, which is likely to affect the assessment of their conservation status (Bickford et al. 2007; Trontelj and Fišer 2008; Hending 2025). This is, however, critical to achieve, in order to set the proper management and conservation actions, and also to foster their inclusion in conservation policies.

Among less studied organisms, lichens harbor several genera which include cryptic species, and not only among the inconspicuous ones, i.e., those with crustose growth form, but even in more conspicuous ones, such as in macrolichens, e.g., in the genera *Letharia* (Altermann et al. 2016), *Parmelia* (Divakar et al. 2005; Corsie et al. 2019; Crespo et al. 2020), *Parmelina* (Nunez-Zapata et al. 2011), *Usnea* (Randlane et al. 2009).

The macrolichen genus *Cetrelia* is a good candidate as a case study in the aforementioned framework, being characterized by morphologically very similar species. *Cetrelia* was established by Culberson and Culberson (1968) to segregate the so-called “parmelioid *Cetrariae*”, broad-lobed foliose epiphytic species with chlorococcoid photobiont, formerly attributed to the genus *Cetraria* Ach., but showing distinct morphological, anatomical and chemical features. Unlike the aforementioned macrolichen genera, which were carefully studied in recent decades, *Cetrelia* has been better known for a longer time in general, albeit being locally understudied. To date, the genus includes 18 species distributed worldwide, with the highest diversity reported from Asia (Randlane and Saag 1991; Mark et al. 2019; Farkas et al. 2021). Five morphotypes, based on the regular presence/type of vegetative propagules, apothecia and/or pseudocyphellae, and six chemotypes, based on the regular occurrence of a specific combination of secondary metabolites (alectoronic and α-collatolic acids; microphyllinic acid; olivetoric acid; anziaic acid; perlatolic acid; imbricaric acid), have been recognized within the genus (Culberson and Culberson 1968; Randlane and Saag 1991).

In Europe, the genus is represented by four species, i.e., *C.cetrarioides* (Duby) W.L. Culb. & C.F. Culb., *C.chicitae* (W.L. Culb.) W.L. Culb. & C.F. Culb., *C.monachorum* (Zahlbr.) W.L. Culb. & C.F. Culb. and *C.olivetorum* (Nyl.) W.L. Culb. & C.F. Culb. All belong to the sorediate morphotype, but each one to a different chemotype: alectoronic and α-collatolic acids (*C.chicitae*), olivetoric acid (*C.olivetorum*), perlatolic acid (*C.cetrarioides*) and imbricaric acid (*C.monachorum*) (Culberson and Culberson 1968; Randlane and Saag 1991). The European *Cetrelia* species can be separated by means of fine morphological characters, e.g., position, shape and size of soralia and pseudocyphellae (Obermayer and Mayrhofer 2007; Farkas et al. 2021), which, however, can often be tricky to interpret due to high intraspecific variability. Thus, they can be identified with more ease and certainty by using their secondary chemistry, by means of a thin-layer chromatography (Orange et al. 2010), since they produce distinct and well-recognizable compounds as main secondary metabolites (Randlane and Saag 1991; Mark et al. 2019; Farkas et al. 2021). Besides their similar morphology, they share also ecological requirements, with different species often growing together in the same sites (Obermayer and Mayrhofer 2007; Howland and Lendemer 2023), which, however, does not explain why some of them are more widespread, whereas others are found more rarely.

**Figure 1.**
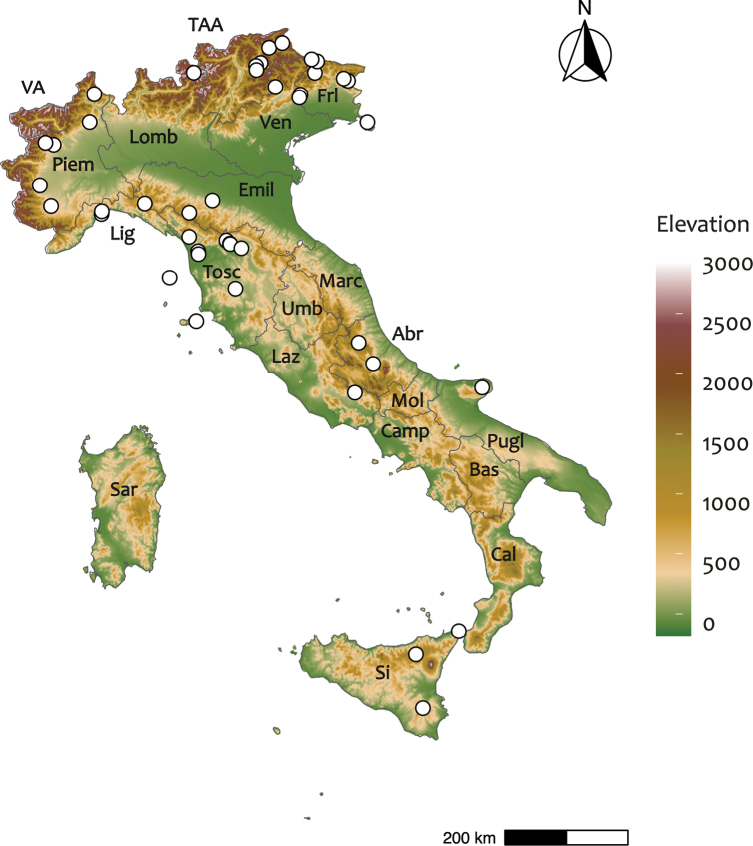
Distribution of the historical records of *Cetrelia* in Italy, mostly under the name *Cetreliaolivetorum* (or earlier synonyms). Frl: Friuli Venezia Giulia; Ven: Veneto; TAA: Trentino Alto Adige; Lomb: Lombardia; Piem: Piemonte; VA: Val d’Aostra; Lig: Liguria; Emil: Emilia Romagna; Tosc: Toscana; Marc: Marche; Umb: Umbria; Laz: Lazio; Abr: Abruzzo; Mol: Molise; Camp: Campania; Pugl: Puglia; Bas: Basilicata; Cal: Calabria; Sar: Sardegna; Sic: Sicilia.

In the last decade, following the revision by Obermayer and Mayrhofer (2007), a few records of *Cetreliacetrarioides*, *C.chicitae* and *C.monachorum* were reported from some Italian regions (see literature cited by Nimis and Martellos 2025), but all the historical records of *Cetrelia* lichens in Italy are under the name *Cetreliaolivetorum* (or its earlier synonyms; Fig. 1). However, this species has been reported as one of the rarest *Cetrelia* in other European countries, e.g., Austria (Obermayer and Mayrhofer 2007), Belarus (Bely et al. 2014; Golubkov et al. 2015), Great Britain (Harrold et al. 2009), Hungary (Farkas et al. 2021), Latvia (Degtjarenko and Moisejevs 2020), Lithuania (Kukwa and Motiejūnaitė 2012), Poland (Kukwa et al. 2012), and the European part of the former Soviet Union (Randlane and Saag 1991). In the light of this updated and more reliable knowledge, it was unlikely that it would be that widespread in Italy, which suggested the need to study the actual distribution of the species belonging to this genus more in depth.

The aim of this study is therefore to provide an updated overview of the genus *Cetrelia* in Italy, resolving the ambiguity left by the older Italian records that was dragged for decades in lichenological literature, and understanding the real distribution of the four species occurring on the national territory. This is crucial also for conservation purposes: the only red list assessment carried out in Italy so far (Nascimbene et al. 2013a) highlighted the poor knowledge of the genus, assigning *C.olivetorum* to “near threatened” and *C.cetrarioides* and *C.monachorum* to “data deficient”. An updated knowledge would allow a more rigorous assessment and provide a stout support for their inclusion in conservation policies, not only at the national level, but also in a European perspective.

## ﻿Materials and methods

### ﻿Sampling and identification

All the specimens referable to *Cetrelia* collected in Italy found in the main European herbaria were retrieved to be revised. In addition, some of the authors carried out new field surveys specifically to contribute to this study with freshly collected material, exploring both localities where *Cetrelia* species were already reported and new localities where, based on habitat types, the occurrence of these species was likely, or at least possible. To increase the probability of collecting all possible species in the surveyed areas, field sampling was carried out by extensive walks, conducted by experts, covering almost all areas where the species could potentially grow. This resulted in the collection of 320 specimens: 46 from 7 public herbaria (FI, GE, GZU, LD, LI, SI, TSB) and 274 from new field explorations, that are currently housed in 5 private herbaria (Benesperi, Di Nuzzo, Gheza, Nascimbene, Pistocchi).

Although morphological differences occur between the four European *Cetrelia* species, chemical characters are considered as the most reliable to distinguish them (Obermayer and Mayrhofer 2007; Farkas et al. 2021; Howland and Lendemer 2023); therefore, our specimens were identified based on chemical characters. Thin-layer chromatography (TLC) was carried out with solvents A, B or B’, and C, according to the method by Orange et al. (2010), to study the main secondary metabolites.

It is not infrequent for specimens belonging to different species to grow intermixed; in some cases, mismatches between chemistry and the morphology of what seemed the only thallus revealed the occurrence of lobes of a different species within the same specimen.

### ﻿DNA extraction, PCR amplification and sequencing

A subset of the specimens was subject to molecular analysis to compare the resulting phylogenetic tree with results already available in the literature (Mark et al. 2019). DNA corresponding to 61 selected samples was extracted from freshly collected material within 24 months from collection. For each sample, a section was taken from the youngest, most peripheral, viable areas of the thallus lobes. Approximately 5 to 7 mg of this material was mechanically lysed using a Ribolyser. DNA extraction was conducted with the InviSorb® Spin Plant Mini Kit (Invitek Diagnostics) according to the manufacturer’s instructions.

Three genomic markers were amplified: the entire internal transcribed spacer region (ITS), partial intergenic spacer region (IGS) from the nuclear ribosomal cistron, and fragments from a putative single-copy protein-coding genes, MCM7. Undiluted DNA extracts were subjected to PCR (polymerase chain reactions) using primers ITS1 and ITS4 (White et al. 1990), IGSf and IGSr (Wirtz et al. 2008), LecMCM7f-LecMCM7r (Leavitt et al. 2011) in a reaction volume of 50 µl. Amplification of the ITS was carried out with illustra™ PuReTaq Ready-To-Go™ (Cytiva) while Amplification of IGS and MCM7 was carried out using DreamTaq DNA Polymerase (ThermoFisher Scientific).

PCR cycling parameters used for amplifying the ITS and IGS regions were: initial denaturation 94 °C for 3 min, followed by 34 cycles of 94 °C for 45 s, 55 °C for 45 s, 72 °C for 1 min, and final elongation 72 °C for 5 min, after which the reaction was cooled to a constant 4 °C. Following Schmitt et al. (2009)PCR cycling parameters used for amplifying the MCM7 region were: initial denaturation 94 °C for 10 min, followed by 38 cycles of 94 °C for 45 s, 56 °C for 50 s, 72 °C for 1 min, and final elongation 72 °C for 5 min.

Electrophoresis confirmed the presence of distinct PCR bands, and all successful products were sent to the Bio-Fab Research laboratory for purification and subsequent Sanger sequencing.

### ﻿Phylogenetic analyses

Sequencing reads were manually verified and edited using FinchTV Version 1.5.0 (Geospiza Research team), with low-quality sequences at both ends removed.

These sequences were compared with the public sequence database using the BLASTn searches and the megaBLAST algorithm against the NCBI database in order to verify their identity to *Cetrelia* and check for any contamination. To root the phylogenetic analyses, 3 sequences respectively for ITS, IGS and MCM7 (KX685872; KX685840; KX685799) and corresponding to *Xanthoparmeliaconspersa* isolate XAC01 were downloaded from GenBank and selected as the outgroup, as recommended by Mark et al. (2019) and Divakar et al. (2015). The sequences were aligned in the program MAFFT v7 (Katoh and Standley 2013) and default parameters (“auto”) for ITS, IGS and MCM7 were selected. Once aligned, the individual marker alignments were concatenated with MEGA11 (Tamura et al. 2021). Bayesian analyses of the concatenated dataset were carried out in MrBayes.v3.3.7a (Huelsenbeck and Ronquist 2001) via the CIPRES Science Gateway (Miller et al. 2010). Best-fit models of nucleotide substitution were determined to be a SYM + G for ITS dataset, TIM2 + G for IGS dataset and K80 + G for MCM7, by the Akaike Information Criterion using jModelTest 2.1.10 (Kalyaanamoorthy et al. 2017). We performed the Bayesian analysis by integrating our sequences with those of *C.monachorum*, *C.olivetorum*, *C.chicitae*, and *C.cetrarioides* as utilized by Mark et al. (2019) and available on GenBank (Sayers et al. 2022). We defined a new matrix with 72 taxa and 1668 characters and performed four independent runs with four chains. Chains were run for 10,000,000 generations each, while parameters and trees were sampled every 1,000^th^ generation. The resulting phylogenetic trees were visualized with FigTree v1.4.4 (Rambaut 2009).

## ﻿Results

### ﻿General results

We retrieved and identified or revised a total of 320 specimens from public herbaria (n = 46: 21 TSB, 7 GE, 7 GZU, 6 FI, 2 LI, 2 SI, 1 LD) and new field collections currently housed in private herbaria (n = 274: 183 herb. Nascimbene, 67 herb. Gheza, 19 herb. Benesperi, 3 herb. Di Nuzzo, 2 herb. Pistocchi) (Suppl. material 1). These specimens were collected in a total of 58 sites (Suppl. material 2).

Despite almost all the old specimens hosted in Italian public herbaria were labelled as “*Cetreliaolivetorum*”, only 4 of them (2 FI and 2 TSB) were confirmed as *C.olivetorum*, the other 21 being referable to *C.monachorum* (n = 15: 7 TSB, 3 GE, 3 FI, 2 SI) and *C.cetrarioides* (n = 6: 3 TSB, 2 GE, 1 FI). Other specimens were labelled correctly, i.e., *C.cetrarioides* (n = 8, TSB) and *C.monachorum* (n = 1, GE), or incorrectly, but not as “*C.olivetorum*” (i.e., 1 “C.cf.chicitae” GE and 1 “*C.cetrarioides*” TSB revealed both to be the real *C.olivetorum*). Specimens hosted in foreign herbaria had been already revised following a TLC-based approach, revealing the occurrence of *C.cetrarioides* (n = 5: 3 GZU, 1 LD, 1 LI) and *C.monachorum* (n = 5: 4 GZU, 1 LI).

Newly collected specimens, almost all collected and identified expressly for this study, referred to all the four species, with *C.monachorum* as the most represented one (n = 162: 119 herb. Nascimbene, 21 herb. Gheza, 19 herb. Benesperi, 3 herb. Di Nuzzo), followed by *C.cetrarioides* (n = 89: 54 herb. Nascimbene, 33 herb. Gheza, 2 herb. Pistocchi); less represented were *C.chicitae* (n = 16: 10 herb. Gheza, 6 herb. Nascimbene) and *C.olivetorum* (n = 6: 4 herb. Nascimbene, 2 herb. Gheza).

### ﻿Distribution of *Cetrelia* species in Italy

*Cetrelia* species were recorded from nine administrative regions (Table 1). All the four European species were found in Friuli Venezia Giulia, Veneto, Trentino Alto Adige, and Lombardia. Two species – *C.monachorum* and *C.olivetorum* s. str. – were found in Liguria and Emilia Romagna, whereas only one species was found in Piemonte (*C.cetrarioides*), Toscana (*C.monachorum*), and Sardegna (*C.monachorum*).

**Table 1.** Overview of the genus *Cetrelia* in Italy. Number of specimens (first number) and number of sites (second number) are reported for each species in each administrative region. Note that the number of sites per region and per species is not necessarily the sum of the number of sites of all the species/regions, since in many sites more than one species occurred.

	*C.cetrarioides*	*C.chicitae*	*C.monachorum*	*C.olivetorum*	No. species	No. specimens	No. sites
**Friuli Venezia Giulia**	23 ; 11	4 ; 2	21 ; 6	2 ; 2	4	50	11
**Veneto**	25 ; 7	2 ; 1	75 ; 11	3 ; 1	4	105	12
**Trentino Alto Adige**	27 ; 14	1 ; 1	47 ; 8	2 ; 2	4	77	16
**Lombardia**	28 ; 6	9 ; 1	12 ; 5	3 ; 2	4	52	8
**Piemonte**	5 ; 2	–	–	–	1	5	2
**Liguria**	–	–	5 ; 2	1 ; 1	2	6	2
**Emilia Romagna**	–	–	2 ; 2	1 ; 1	2	3	3
**Toscana**	–	–	20 ; 3	–	1	20	3
**Sardegna**	–	–	1 ; 1	–	1	1	1
**No. Regions**	5	4	8	6			
**No. Specimens**	108	16	183	12		319	
**No. Sites**	40	5	38	9			58

### ﻿Phylogenetic analyses

A total of 51 new sequences were amplified for ITS region, 35 for IGS region and 22 for MCM7 region. All the four *Cetrelia* species analyzed in this study are represented in these sequences (Suppl. material 3). BLAST searches on GenBank revealed some conflicting results between TLC-based and molecular determination in 8 cases (Suppl. material 3), due to the aforementioned occurrence of specimens including mixed lobes of different species. The phylogenetic tree (Suppl. material 4) reveals a clade structure consistent with the findings of Mark et al. (2019). The nodes corresponding to the four species groups are well-supported. *Cetreliamonachorum* diverges early as a monophyletic group (posterior probability PP = 1), while the second node (PP = 1) positions the *C.olivetorum* group as a sister to the clade (PP = 1) that confirms the close relationship between *C.cetrarioides* and *C.chicitae*.

### ﻿Taxonomy

Based on the morphological and chemical comparison of the collected specimens, supported by DNA analyses, we provide an updated description of the morphology and chemical profile for each species. Additionally, we summarize information on their distribution, habitat, and phorophytes in Italy, also commenting their past literature records in the country. An overview of the main diagnostic morphological characteristics is given in Table 2.

**Table 2.** Overview of the main diagnostic morphological characters between the Italian species of *Cetrelia*, intended for well-developed, typical specimens; intraspecific variability is high, therefore exceptions are often possible. For further details see Obermayer and Mayrhofer (2007).

	*C.cetrarioides*	*C.chicitae*	*C.monachorum*	*C.olivetorum*
**Pseudocyphellae on the upper surface of the lobes**	Small to large, flat or rarely raised; often lacking from the central part of the thallus.	Large, flat; usually also in the central part of the thallus.	Small, raised, slightly convex; often lacking from the central part of the thallus.	Small, flat or slightly concave/immersed; often lacking from the central part of the thallus.
**Pseudocyphellae on the lower surface of the lobes**	Small to large, flat on sterile lobes; small, raised, slightly convex on fertile lobes; generally common.	Small, raised, slightly convex, very rare.	Small, raised, slightly convex, very rare.	Small, raised, slightly convex, very rare.
**Soredia**	Fine, 25–35[-40] μm.	Coarse, [35-]40–55 μm.	Coarse, [35-]40–55 μm.	Fine or coarse, 25–55 μm.
**Marginal soralia**	Smooth, convex, labriform.	Strongly twisted-undulate, with a crenulate appearance.	Irregular.	Smooth, convex, labriform.
**Overall appearance**	Fine soredia in smooth, convex marginal soralia and small to large, flat pseudocyphellae on the lower surface of sterile lobes.	Coarse soredia in strongly twisted-undulate marginal soralia and large, flat pseudocyphellae generally only on the upper surface.	Coarse soredia in irregular marginal soralia and raised pseudocyphellae on the upper surface.	Fine (but coarser than in *C.cetrarioides*) or coarse soredia in smooth, convex marginal soralia and small, flat to concave pseudocyphellae on the upper surface.

#### ﻿ Cetrelia cetrarioides

Taxon classificationFungiLecanoralesParmeliaceae

(Duby) W.L. Culb. & C.F. Culb.

F1722AB9-7A6E-540C-A658-DB583B08EFAC

##### Description.

Thallus foliose, heteromerous, dorsiventral, loosely attached, forming wavy, wide-spreading, usually orbicular, wide patches. Upper surface greenish-grey, lower surface black in the central part to brown at the lobe edges. Lobes broad and rounded, up to 20 mm wide, with raised margins. Pseudocyphellae on upper surface punctiform, small to rather large, usually not raised, often lacking in the central parts of thallus; pseudocyphellae on lower surface frequently present, at least on some ascending, contorted lobe apices. Soralia primarily marginal, elongated, usually very smooth and strongly convex, with fine soredia (25–35 µm). Lower surface wrinkled, with scattered, simple, black rhizines and a rhizine-free zone along the margin. Apothecia lecanorine, with brown disc. Found fertile only once in Italy (Piemonte: Val Anzasca).

##### Chemistry.

Cortex with atranorin (sometimes in low concentrations); medulla with perlatolic acid (major), imbricaric acid (minor or absent), anziaic acid (traces or absent).

##### Distribution in Italy.

**Figure 2.**
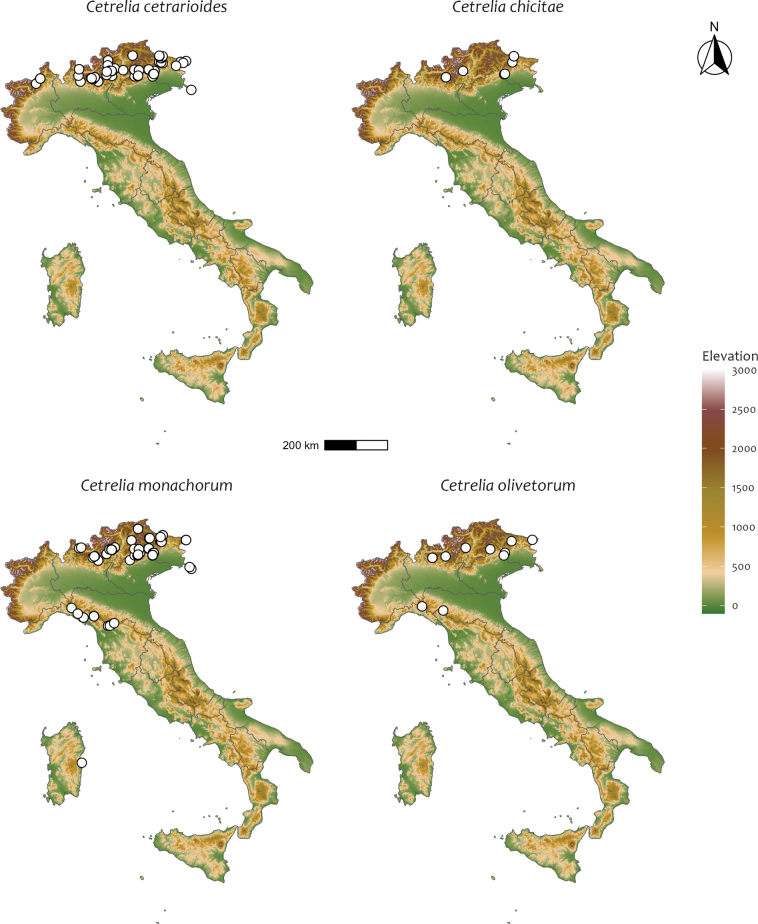
Updated distribution of the four *Cetrelia* species in Italy.

All of the Alps (108 specimens from 40 sites), but more frequent in the central-eastern part: Friuli Venezia Giulia (23 specimens from 11 sites), Veneto (25 specimens from 7 sites), Trentino Alto Adige (27 specimens from 14 sites), Lombardia (28 specimens from 6 sites), Piemonte (5 specimens from 2 sites). Fig. 2.

##### Habitat.

Beech, gray alder, coniferous or mixed beech-coniferous forests in the montane belt (400–1450 m a.s.l.).

##### Phorophytes.

*Abiesalba*, *Acerpseudoplatanus*, *Corylusavellana*, *Fagussylvati*ca, *Fraxinusexcelsior*, *Larixdecidua*, *Piceaabies*, *Quercuspubescens*, *Salixappendiculata*, *Salixcaprea*, *Salix* sp., *Ulmus* sp.

##### Literature.

***Confirmed citations***: Veneto: Obermayer and Mayrhofer (2007), Nascimbene et al. (2010); Trentino Alto Adige: Obermayer and Mayrhofer (2007), Lang et al. (2010), Nascimbene (2014), Nascimbene and Marini (2015), Trindade et al. (2021), Nascimbene et al. (2022); Lombardia: Gheza (2019), Ravera et al. (2021). *Erroneous citations* (specimens belonging to other species): Emilia Romagna: Tretiach et al. (2008) (*C.olivetorum*), Fariselli et al. (2020) (*C.olivetorum*).

##### Specimens examined.

See Suppl. material 1.

#### ﻿ Cetrelia chicitae

Taxon classificationFungiLecanoralesParmeliaceae

(W.L. Culb.) W.L. Culb. & C.F. Culb.

41B0F02A-6114-526D-AF09-987DAD94F0BF

##### Description.

Thallus foliose, heteromerous, dorsiventral, loosely attached, forming wavy, wide-spreading, usually orbicular, wide rosettes. Upper surface glaucous-grey, lower surface black in the central part to brown at the lobe edges. Lobes broad and round, up to 20 mm wide, with raised margins. Pseudocyphellae punctiform to irregularly-shaped on upper surface, which are rather large and usually not raised, also developed in the older, central parts of thallus; pseudocyphellae on lower surface not developed, or appearing as very small white dots. Soralia primarily marginal on strongly twisted lobes, giving the lobe-ends a somewhat nibbled appearance, usually smooth, with coarse soredia (35–55 µm). Lower surface rather regularly ridged, with scattered, simple, black rhizines and a rhizine-free zone along the margin. Never found fertile in Italy.

##### Chemistry.

Cortex with atranorin (sometimes in low concentrations); medulla with alectoronic, α-collatolic and physodic acids.

##### Distribution in Italy.

Central-eastern Alps (16 specimens from 5 sites): Friuli Venezia Giulia (4 specimens from 2 sites), Veneto (2 specimens from 1 sites), Trentino Alto Adige (1 specimen from 1 site), Lombardia (9 specimens from 1 site). Fig. 2.

##### Habitat.

Beech, coniferous or mixed beech-coniferous forests in the montane belt (870–1195 m a.s.l.), moist due to frequent rainfall and/or fog, usually near streams.

##### Phorophytes.

*Abiesalba*, *Fagussylvati*ca.

##### Literature.

***Confirmed citations***: Friuli Venezia Giulia: Nascimbene et al. (2021); Veneto: Nascimbene et al. (2021).

##### Remarks.

This is the rarest *Cetrelia* species in Italy, which is in accord with the data from the rest of Europe (Obermayer and Mayrhofer 2007; Harrold et al. 2009). It is instead one of the most widespread species in other areas, e.g., North America (Howland and Lendemer 2023) and India (Mishra and Upreti 2015).

##### Specimens examined.

See Suppl. material 1.

#### ﻿ Cetrelia monachorum

Taxon classificationFungiLecanoralesParmeliaceae

(Zahlbr.) W.L. Culb. & C.F. Culb.

8895B240-4A80-5871-995B-1814515DE91E

##### Description.

Thallus foliose, heteromerous, dorsiventral, loosely attached, forming wavy, wide-spreading, usually orbicular, wide rosettes. Upper surface greenish-grey, lower surface black in the central part to brown at the lobe edges. Lobes broad and round, up to 20 mm wide, with raised margins. Pseudocyphellae on upper surface frequently developing on slightly raised structures, often appearing as accumulation of individuals to form a larger unit, sometimes lacking in the central parts of older thalli; absent from lower surface. Soralia primarily marginal, often rather irregularly shaped, with coarse soredia (35–55 µm). Lower surface wrinkled, with scattered, simple, black rhizines and a rhizine-free zone along the margin. Apothecia lecanorine, with brown disc. Found fertile three times in Italy (Trentino Alto Adige: Val Brenta, 1; Veneto: Cansiglio, 2).

##### Chemistry.

Cortex with atranorin (sometimes in low concentrations); medulla with imbricaric acid (major), perlatolic acid (minor or absent), anziaic acid (traces or absent).

##### Distribution in Italy.

Central-eastern Alps (155 specimens from 30 sites): Friuli Venezia Giulia (21 specimens from 6 sites), Veneto (75 specimens from 11 sites), Trentino Alto Adige (47 specimens from 8 sites), Lombardia (12 specimens from 5 sites); Northern Apennines (27 specimens from 7 sites): Liguria (5 specimens from 2 sites), Emilia Romagna (2 specimens from 2 sites), Toscana (20 specimens from 3 sites); Sardegna (1 specimen from 1 site). Fig. 2.

##### Habitat.

Beech, coniferous or mixed beech-coniferous forests and chestnut groves from the hilly to the montane belt (274–1600 m a.s.l.).

##### Phorophytes.

*Abiesalba*, *Acerpseudoplatanus*, *Alnusincana*, *Castaneasativa*, *Corylusavellana*, *Fagussylvati*ca, *Fraxinusexcelsior*, *Piceaabies*, *Prunusavium*, *Quercuscerris*, *Salixcaprea*, *Salix* sp.

##### Literature.

***Confirmed citations***: Friuli Venezia Giulia: Obermayer and Mayrhofer (2007); Veneto: Nascimbene et al. (2021); Trentino Alto Adige: Obermayer and Mayrhofer (2007), Nascimbene (2014), Nascimbene and Marini (2015), Trindade et al. (2021), Nascimbene et al. (2022); Liguria: Ravera et al. (2019); Sardegna: Nascimbene et al. (2021).

##### Remarks.

The record of “*C.olivetorum*” by Brackel (2015) should be referred to this species, since the only *Cetrelia* species found by us in the same site was *C.monachorum*.

##### Specimens examined.

See Suppl. material 1.

#### ﻿ Cetrelia olivetorum

Taxon classificationFungiLecanoralesParmeliaceae

(Nyl.) W.L. Culb. & C.F. Culb.

81492FE2-5218-5BDD-974B-00FA23BE2332

##### Description.

Thallus foliose, heteromerous, dorsiventral, loosely attached, forming wavy, wide-spreading, usually orbicular, wide rosettes. Upper surface greenish-gray to glaucous-gray, lower surface black in the central part to brown at the lobe edges. Lobes broad and round, to 20 mm wide, with raised margins. Pseudocyphellae punctiform on upper surface, which are rather large and usually not raised, also developed in the older, central parts of thallus; pseudocyphellae on the lower surface not developed, or appearing as very small white dots. Soralia primarily marginal on strongly twisted lobes, giving the lobe-ends a somewhat nibbled appearance, usually smooth, with coarse soredia (25–55 µm). Lower surface rather regularly ridged, with scattered, simple, black rhizines and a rhizine-free zone along the margin. Never found fertile in Italy.

##### Chemistry.

Cortex with atranorin (sometimes in low concentrations); medulla with olivetoric acid.

##### Distribution in Italy.

Central-eastern Alps (10 specimens from 7 sites): Friuli Venezia Giulia (2 specimens from 2 sites), Veneto (3 specimens from 1 site), Trentino Alto Adige (2 specimens from 2 sites), Lombardia (3 specimens from 2 sites); Northern Apennines (2 specimens from 2 sites): Liguria (1 specimen from 1 site), Emilia Romagna (1 specimen from 1 site). Fig. 2.

##### Habitat.

Beech or mixed beech-coniferous forests in the montane belt (650–1450 m a.s.l.), moist due to frequent rainfall and/or fog, often near waterbodies (streams, lakes).

##### Phorophytes.

*Abiesalba*, *Acerpseudoplatanus*, *Castaneasativa*, *Fagussylvati*ca, *Piceaabies*.

##### Literature.

***Confirmed citations***: Friuli Venezia Giulia: Nimis (1982). ***Dubious citations*** (specimens not found): Friuli Venezia Giulia: Glowacki (1874), Clerc (1984), Carvalho (1997); Veneto: Du Rietz (1924), Lazzarin (1997), Caniglia et al. (1999), Thor and Nascimbene (2007), Brackel (2013); Trentino Alto Adige: Lettau (1957), Caniglia et al. (1988), Nascimbene and Caniglia (2000), Nascimbene (2005a, 2006, 2008a), Stofer (2006), Nascimbene et al. (2007, 2022), Lang et al. (2010), Nimis et al. (2015); Lombardia: Stizenberger (1882), Du Rietz (1924); Piemonte: Jatta (1909/11), Martel (1910), Montacchini and Piervittori (1979), Griselli et al. (2003); Emilia Romagna: Zanfrognini (1902), Nimis (1984, 1985), Fariselli et al. (2020); Toscana: Baglietto (1871), Sambo (1927); Abruzzo: Jatta (1909/11), Recchia and Villa (1996), Stofer (2006), Gheza et al. (2021); Puglia: Nimis and Tretiach (1999); Sicilia: Marino and Paratore (1900), Jatta (1909/11), Grillo and Caniglia (2004, 2006). ***Erroneous citations*** (specimens belonging to other species): Friuli Venezia Giulia: Nimi and Loi (1984) (*C.monachorum*), Tretiach and Molaro (2007) (*C.cetrarioides*); Veneto: Nascimbene and Caniglia (1997, 2002, 2003), Nascimbene (2005b, 2008b, 2011), Nascimbene et al. (2005, 2006, 2007, 2009, 2010, 2013b) (all *C.cetrarioides* and *C.monachorum*); Trentino Alto Adige: Kernstock (1890) (*C.monachorum*); Lombardia: Jatta (1909/11) (*C.monachorum*); Piemonte: Baglietto and Carestia (1865, 1880) (*C.cetrarioides*), Isocrono et al. (2004) (*C.cetrarioides*); Liguria: Putortì et al. (1999) (*C.monachorum*), Giordani and Brunialti (2000) (*C.monachorum*), Brunialti et al. (2001) (*C.monachorum*); Toscana: Brackel (2015) (*C.monachorum*).

##### Remarks.

We were not able to retrieve duplicates of the *exsiccata* distributed by Anzi as Lichenes Rariores Langobardiae n. 48 and Lichenes Minus Rari Italiae Superioris n. 99, cited by Stizenberger (1882) and Du Rietz (1924). The record by Kernstock (1890) likely refers to specimens distributed as Flora Exsiccata Austro-Hungarica n. 3117, of which we revised 5 specimens (GB-0178237, LD-1086559, and three unnumbered specimens from GZU), all referring to *C.monachorum*. About the records of “*C.olivetorum*” by Baglietto and Carestia (1865, 1880), that were cited also by Isocrono et al. (2004), we were able to retrieve just one *exsiccatum* (LD-1064112) that refers to *C.cetrarioides*.

##### Specimens examined.

See Suppl. material 1.

## ﻿Discussion

All the four European species of *Cetrelia* currently occur in Italy, but with different ranges. The most widespread species is *C.monachorum*, which was found in 38 sites, ranging from the Alps to the Apennines, and occurring also in one site in Sardegna. *C.cetrarioides* is widespread as well, occurring in 40 sites, but being limited to the Alps. *C.olivetorum* shows scattered occurrences in the central-eastern Alps and the northern Apennines, in a total of nine sites. Finally, *C.chicitae* is confined to a few very well-preserved moist coniferous-beech forest stands in five sites in the central-eastern Italian Alps.

Only three sites, i.e., Pista degli Abeti in Val di Scalve (Lombardia), Foresta del Cansiglio (Veneto) and Lago di Sauris (Friuli Venezia Giulia), hosted all the four *Cetrelia* species together, whereas five sites (Foresta di Tarvisio and Val Fleons in Friuli Venezia Giulia; Lago di Tovel, Val Brenta and Val Canali in Trentino Alto Adige) hosted three species. In all the other 50 sites, only either two or one species were found. The three sites hosting all the four *Cetrelia* species are remarkable for their high environmental quality and ecological continuity, which reflect in the high number of other rare and/or threatened macrolichens occurring there (Suppl. material 5). This is in accord with what has been observed also in North America (Howland and Lendemer 2023). *Cetreliachicitae*, in particular, has always been found related to high-quality forest habitats (Obermayer and Mayrhofer 2007; Howland and Lendemer 2023).

In a very few sites we collected fertile material of *C.cetrarioides* (one specimen in one site in Piemonte) and *C.monachorum* (one specimen in one site in Trentino Alto Adige and two specimens in one site in Veneto). Fertile specimens have been reported very rarely in contemporary literature (Obermayer and Mayrhofer 2007), and apothecia are likely produced only in highly optimal habitat conditions.

Most of our records of all the four Italian *Cetrelia* species are from well-preserved, moist forests dominated by conifers and/or beech, located in the montane belt of the Alps and Apennines. Their ecology seems similar, as observed also in other geographical areas (Obermayer and Mayrhofer 2007; Kukwa et al. 2012; Farkas et al. 2021), but differences in distribution suggest that there are also slight differences in ecological preferences, e.g., some species perhaps need more moisture. *Cetreliachicitae*, the rarest species, occurs only in very well-preserved forests, in some cases even managed (i.e., Pista degli Abeti and Cansiglio), but granting very long ecological continuity and habitat stability, since the persistence of such forests has been documented for at least three centuries. The occurrence of *Cetrelia* species, namely *C.monachorum* and *C.olivetorum*, in the Northern Apennines seems to be restricted to chestnut groves in the lower montane belt. In that area, this anthropogenic forest type occurs in moist sites and is characterized by a long ecological continuity, also hosting interesting epiphytic assemblages rich in species of conservation concern (Matteucci et al. 2012).

The confusion in Italian herbarium material previous to this revision, with most of the specimens misidentified or placed under the rough name of “*Cetreliaolivetorum*”, also after the recognition that different chemotypes belong to different species (Obermayer and Mayrhofer 2007), suggests that morphology and spot test results alone are often not diagnostic enough to identify *Cetrelia* specimens at the species level with a satisfying degree of certainty. Therefore, TLC should always be carried out when dealing with this genus, since secondary chemistry is always diagnostic and backed by molecular data (Obermayer and Mayrhofer 2007; Mark et al. 2019; Farkas et al. 2021; Howland and Lendemer 2023). A few morphological characters can be diagnostic in well-developed specimens (Table 2; see also the key by Obermayer and Mayrhofer 2007). Well-developed pseudocyphellae on upwardly curved marginal areas of the underside of the thallus, in combination with smooth soralia, can be found only in *C.cetrarioides*. Strongly twisted soralia, in combination with very coarse soredia and big, flat pseudocyphellae, only in *C.chicitae*. Distinctly raised pseudocyphellae, in combination with very coarse-grained soralia, only in *C.monachorum*. Finally, a C+ clearly red reaction of the medulla, in combination with smooth soralia, is found only in *C.olivetorum*.

Further elements that contribute to complicate the assessment of the actual rarity of *Cetrelia* species are the slightly different microhabitat preferences, which were observed in the field on some occasions. For example, *C.chicitae* was found very rarely on trunks, more often on branches and twigs; the real distribution of species limited to, or preferentially growing in the canopy can be very difficult to assess reliably (Rosso et al. 2000).

Red list assessment in particular risks to be negatively affected by the missed recognition of the species. The preliminary Red List of Italian epiphytic lichens (Nascimbene et al. 2013a), carried out with a simplified approach, included the three species reported from Italy at the time. Unfortunately, the assessment of *C.olivetorum* (evaluated as “Near-threatened”) was affected by the amount of erroneous literature records, whereas the assessment of *C.cetrarioides* and *C.monachorum*, reported from Italy since a few years, was not possible due to the scarcity of data, and both were evaluated as “Data Deficient”. A red list assessment has not been undertaken in the present work, mainly due to the impossibility of making a comparison with reliable historical data. Other European countries were able to assess *Cetrelia* species according to the IUCN criteria. In Estonia, *C.cetrarioides* and *C.olivetorum* were evaluated as “Endangered”, and *C.monachorum* as “Critically Endangered” (Lõhmus et al. 2019). In Latvia, *C.cetrarioides* and *C.olivetorum* were evaluated as “Vulnerable”, and *C.monachorum* as “Endangered” (Degtjarenko et al. 2024). In general, the trend seems to indicate that these species are indeed at risk in Europe.

Unfortunately, we were not able to verify several historical literature records from southern regions, i.e., those from Abruzzo (Jatta 1909–1911; Recchia and Villa 1996; Stofer 2006), Puglia (Nimis and Tretiach 1999) and Sicilia (Marino and Paratore 1900; Jatta 1909–1911; Grillo and Caniglia 2004, 2006), since specimens referred to those records were not retrieved from any herbarium. Albeit at least some of those records seem reliable, provided the caveat of considering them as “*Cetrelia* sp.” and not “*Cetreliaolivetorum* s. str.”, they are mostly old, therefore making the current occurrence of *Cetrelia* spp. in southern Italy at least dubious. The fact that *Cetrelia* specimens were not found even in potentially suitable biotopes visited recently by some of us, e.g., the Abetina di Rosello (Nascimbene and Pellegrini 2021) in Abruzzo, support this theory.

## ﻿Conclusions

All the four *Cetrelia* species reported from Italy and Europe so far occur in old, moist forests dominated by beech and/or conifers in the montane belt, but they show different biogeographical patterns. Two of them, i.e., *C.cetrarioides* and *C.monachorum*, are distributed more widely and are more abundant in their occurrence sites, whereas the other two, i.e., *C.chicitae* and *C.olivetorum*, have a less broad and more scattered distribution and are usually not as abundant as the previous ones. Furthermore, two species, i.e., *C.cetrarioides* and *C.chicitae*, are limited to the Alps, whereas the other two, i.e., *C.monachorum* and *C.olivetorum*, reach also the northern Apennines. Protection should be advisable for the sites hosting all the four species together, also in consideration of the other species of conservation interest co-occurring with them in those sites. All this should be carefully considered when planning both forest management and conservation actions aimed at including lichens at the local and the national scales.

Elucidating the real current distribution of *Cetrelia* species in Italy is a first step to better understand their biogeography and their ecology, which is needed to include them in conservation policies. In this context, we emphasize the crucial role of both herbarium collections and targeted fieldwork in assessing species distribution. Herbarium collections not only can provide a starting point for selecting survey sites, but also allow for direct comparisons between newly collected samples and historical specimens. This approach provides a more comprehensive and reliable distribution assessment than one based solely on field sampling. On the other hand, new fieldwork is crucial, not only to assess the permanence of species in historically known sites, but also to discover new ones and to collect fresh specimens suitable for molecular analyses, which are often not possible on old herbarium material.

Accurate knowledge of species distribution forms the foundation for more applied tasks, and represents a fundamental step, especially for cryptic species, for which obtaining reliable and comprehensive data is challenging compared to more easily identifiable ones. Without a solid grasp of their distribution, conservation strategies may miss vital elements of ecosystem health and biodiversity protection. Also, the protection of cryptic species is challenging, due to the difficulty – often the impossibility – to recognize them at the species level with certainty in the field. While waiting for new techniques to be developed to achieve this, a holistic approach to wide-scale protection and conservation, e.g., based on protected areas networks and area-based conservation, is critical to guarantee the survival of such peculiar components of biodiversity (Hending 2025).

## Supplementary Material

XML Treatment for Cetrelia cetrarioides

XML Treatment for Cetrelia chicitae

XML Treatment for Cetrelia monachorum

XML Treatment for Cetrelia olivetorum

## ﻿Additional information

### Author ORCIDs

Gabriele Gheza https://orcid.org/0009-0009-7524-8522

Chiara Vallese https://orcid.org/0000-0002-8531-5954

Luca Di Nuzzo https://orcid.org/0000-0002-5558-9401

Renato Benesperi https://orcid.org/0000-0003-4296-3393

Elisabetta Bianchi https://orcid.org/0000-0003-1197-4081

Silvia Del Vecchio https://orcid.org/0000-0001-8458-0433

Luana Francesconi https://orcid.org/0000-0003-1745-7069

Paolo Giordani https://orcid.org/0000-0003-0087-7315

Pier Luigi Nimis https://orcid.org/0000-0003-3523-0183

Helmut Mayrhofer https://orcid.org/0000-0002-9433-3847

### Data availability

All of the data that support the findings of this study are available in the main text or Supplementary Information.

## Supplementary materials

Supplementary material 1 Specimens list, occurence and data Data type xlsx Explanation note For each sample, the place and data of collection are provided with coordinates and altitude, the name of the person who collected and identified it, the place where the sample is located (herbarium), chemistry. https://binary.pensoft.net/file/1391035This dataset is made available under the Open Database License (http://opendatacommons.org/licenses/odbl/1.0/). The Open Database License (ODbL) is a license agreement intended to allow users to freely share, modify, and use this Dataset while maintaining this same freedom for others, provided that the original source and author(s) are credited.Gabriele Gheza, Chiara Vallese, Luca Di Nuzzo, Simona Corneti, Renato Benesperi, Elisabetta Bianchi, Giulia Canali, Silvia Del Vecchio, Luana Francesconi, Paolo Giordani, Pier Luigi Nimis, Walter Obermayer, Chiara Pistocchi, Helmut Mayrhofer, Juri Nascimbene

Supplementary material 2 List of collection sites Data type xlsx https://binary.pensoft.net/file/1391036This dataset is made available under the Open Database License (http://opendatacommons.org/licenses/odbl/1.0/). The Open Database License (ODbL) is a license agreement intended to allow users to freely share, modify, and use this Dataset while maintaining this same freedom for others, provided that the original source and author(s) are credited.Gabriele Gheza, Chiara Vallese, Luca Di Nuzzo, Simona Corneti, Renato Benesperi, Elisabetta Bianchi, Giulia Canali, Silvia Del Vecchio, Luana Francesconi, Paolo Giordani, Pier Luigi Nimis, Walter Obermayer, Chiara Pistocchi, Helmut Mayrhofer, Juri Nascimbene

Supplementary material 3 List of the subset of samples subjected to phylogenetic analysis Data type docx Explanation note Data on each sample's collection date, place, and storage is reported, along with the lab's assigned code and markers used. Species names, determined through morphological and chemical analyses and BLAST results, are also provided, with BLAST-based identifications highlighted in bold. Samples marked with an asterisk (*) indicate cases where the phylogenetic analysis results differ from the morphological and chemical findings.Please note that (+) will soon be replaced with GenBank accession numbers. https://binary.pensoft.net/file/1391037This dataset is made available under the Open Database License (http://opendatacommons.org/licenses/odbl/1.0/). The Open Database License (ODbL) is a license agreement intended to allow users to freely share, modify, and use this Dataset while maintaining this same freedom for others, provided that the original source and author(s) are credited.Gabriele Gheza, Chiara Vallese, Luca Di Nuzzo, Simona Corneti, Renato Benesperi, Elisabetta Bianchi, Giulia Canali, Silvia Del Vecchio, Luana Francesconi, Paolo Giordani, Pier Luigi Nimis, Walter Obermayer, Chiara Pistocchi, Helmut Mayrhofer, Juri Nascimbene

Supplementary material 4 Phylogenetic tree Data type pdf https://binary.pensoft.net/file/1391038This dataset is made available under the Open Database License (http://opendatacommons.org/licenses/odbl/1.0/). The Open Database License (ODbL) is a license agreement intended to allow users to freely share, modify, and use this Dataset while maintaining this same freedom for others, provided that the original source and author(s) are credited.Gabriele Gheza, Chiara Vallese, Luca Di Nuzzo, Simona Corneti, Renato Benesperi, Elisabetta Bianchi, Giulia Canali, Silvia Del Vecchio, Luana Francesconi, Paolo Giordani, Pier Luigi Nimis, Walter Obermayer, Chiara Pistocchi, Helmut Mayrhofer, Juri Nascimbene

Supplementary material 5 Macrolichens of conservation interest co-occurring with *Cetrelia* Data type doc Explanation note Macrolichens of conservation interest co-occurring with the four *Cetrelia* species in the only three Italian sites hosting all of them. Nomenclature follows Nimis and Martellos (2025). https://binary.pensoft.net/file/1391039This dataset is made available under the Open Database License (http://opendatacommons.org/licenses/odbl/1.0/). The Open Database License (ODbL) is a license agreement intended to allow users to freely share, modify, and use this Dataset while maintaining this same freedom for others, provided that the original source and author(s) are credited.Gabriele Gheza, Chiara Vallese, Luca Di Nuzzo, Simona Corneti, Renato Benesperi, Elisabetta Bianchi, Giulia Canali, Silvia Del Vecchio, Luana Francesconi, Paolo Giordani, Pier Luigi Nimis, Walter Obermayer, Chiara Pistocchi, Helmut Mayrhofer, Juri Nascimbene
