# Supplementary material for: ﻿Towards a better knowledge and conservation of cryptic macrolichens in Italy: a revision of the genus Cetrelia (Parmeliaceae, Lecanorales, lichenized Ascomycota)
Source: MycoKeys. 2025 Aug 8;120:231–54. doi: 10.3897/mycokeys.120.154233 (PMC12357148; doi:10.3897/mycokeys.120.154233)
Supplement: Supplementary material 3 — List of the subset of samples subjected to phylogenetic analysis [file mycokeys-120-231-s003.docx]

**Towards a better knowledge and conservation of cryptic macrolichens in Italy: a revision of the genus Cetrelia W.L. Culb. & C.F. Culb.**

Gabriele Gheza, Chiara Vallese, Luca Di Nuzzo, Simona Corneti, Renato Benesperi, Elisabetta Bianchi, Giulia Canali, Silvia Del Vecchio, Luana Francesconi, Paolo Giordani, Pier Luigi Nimis, Walter Obermayer, Chiara Pistocchi, Helmut Mayrhofer, Juri Nascimbene

**Supplementary File 3**

Subset of samples subjected to phylogenetic analysis. For each sample, the collection date, location, and storage information are reported, along with the laboratory-assigned code and genetic markers analyzed. Genbank accession numbers for each marker are also provided

| **Laboratory code** | **Species** | **Voucher info  (date; locality; storage)** | **Markers** | | |
| --- | --- | --- | --- | --- | --- |
|  |  |  | **ITS** | **IGS** | **MCM7** |
| ecv- 1 | *Cetrelia monachorum* | 4/2022; Lombardia: Schilpario, Pista degli Abeti; Herbarium Gheza | PV187697 | PV423748 | PV423783 |
| ecv- 5 | *Cetrelia cetrarioides* | 4/2022; Lombardia: Schilpario, Pista degli Abeti; Herbarium Gheza | PV187698 | PV423749 |  |
| ecv- 6 | *Cetrelia monachorum* | 10/2021; Lombardia: Schilpario, Pista degli Abeti; Herbarium Gheza | PV187699 | PV423750 |  |
| ecv- 7 | *Cetrelia olivetorum* | 6/2017; Trentino Alto Adige: Tuenno, Lago di Tovel; Herbarium Gheza | PV187700 | PV423751 |  |
| ecv- 9 | *Cetrelia monachorum* | 8/2022; Trentino Alto Adige: Tre Ville, Val Brenta; Herbarium Gheza | PV187701 |  |  |
| ecv- 10 | *Cetrelia monachorum* | 4/2022, Veneto: Dolomiti Bellunesi Natural Park, Monte Avena; Herbarium Nascimbene (JN7914) | PV187702 | PV423752 | PV423784 |
| ecv- 11 | *Cetrelia cetrarioides* | 4/2022, Veneto: Dolomiti Bellunesi Natural Park, Monte Avena; Herbarium Nascimbene (JN7915) | PV187703 | PV423753 | PV423785 |
| ecv- 13 | *Cetrelia monachorum* | 2/2022; Veneto: Dolomiti Bellunesi Natural Park, Saladen; Herbarium Nascimbene (JN7917) | PV187704 | PV423754 | PV423786 |
| ecv- 15 | *Cetrelia monachorum* | 4/2022; Lombardia: Schilpario, Pista degli Abeti; Herbarium Gheza | PV187705 | PV423755 |  |
| ecv- 16 | *Cetrelia cetrarioides* | 4/2022; Lombardia: Schilpario, Pista degli Abeti; Herbarium Gheza | PV187706 | PV423756 | PV423787 |
| ecv- 17 | *Cetrelia cetrarioides* | 4/2022; Lombardia: Schilpario, Pista degli Abeti; Herbarium Gheza | PV187707 | PV423757 |  |
| ecv- 18 | *Cetrelia cetrarioides* | 4/2022; Lombardia: Schilpario, Pista degli Abeti; Herbarium Gheza | PV187708 |  |  |
| ecv- 19 | *Cetrelia monachorum* | 4/2022; Lombardia: Azzone, Boschi del Giovetto; Herbarium Gheza | PV187709 |  |  |
| ecv- 20 | *Cetrelia cetrarioides* | 4/2022; Lombardia: Azzone, Roccolo del Giovetto; Herbarium Gheza | PV187710 | PV423758 |  |
| ecv- 21 | *Cetrelia cetrarioides* | 4/2022; Lombardia: Azzone, Roccolo del Giovetto; Herbarium Gheza | PV187711 | PV423759 | PV423788 |
| ecv- 22 | *Cetrelia cetrarioides* | 8/2019; Lombardia: Bienno, Val Grigna; Herbarium Gheza | PV187712 | PV423760 |  |
| ecv- 23 | *Cetrelia monachorum* | 8/2019; Lombardia: Bienno, Val Grigna; Herbarium Gheza | PV187713 | PV423761 |  |
| ecv- 24 | *Cetrelia cetrarioides* | 8/2019; Lombardia: Valmasino, Bagni di Masino; Herbarium Gheza | PV187714 | PV423762 |  |
| ecv- 25 | *Cetrelia monachorum* | 8/2019; Lombardia: Valmasino, Val di Mello; Herbarium Gheza | PV187715 | PV423763 |  |
| ecv- 26 | *Cetrelia chicitae* | 8/2022; Trentino Alto Adige: Tre Ville, Val Brenta; Herbarium Gheza | PV187716 | PV423764 | PV423789 |
| ecv- 28 | *Cetrelia cetrarioides* | 8/2022; Trentino Alto Adige: Tre Ville, Val Brenta; Herbarium Gheza | PV187717 |  | PV423790 |
| ecv- 29 | *Cetrelia monachorum* | 8/2022; Trentino Alto Adige: Tre Ville, Val Brenta; Herbarium Gheza | PV187718 | PV423765 | PV423791 |
| ecv- 30 | *Cetrelia monachorum* | 8/2022; Trentino Alto Adige: Tre Ville, Val Brenta; Herbarium Gheza | PV187719 |  |  |
| ecv- 31 | *Cetrelia monachorum* | 8/2022; Trentino Alto Adige: Tre Ville, Val Brenta; Herbarium Gheza | PV187720 |  |  |
| ecv- 32 | *Cetrelia monachorum* | 8/2022; Trentino Alto Adige: Tre Ville, Val Brenta; Herbarium Gheza | PV187721 |  |  |
| ecv- 33 | *Cetrelia monachorum* | na/2022; Toscana: Penna di Lucchio; Herbarium Di Nuzzo | PV187722 |  |  |
| ecv- 34 | *Cetrelia monachorum* | 2/2021; Toscana: Piteglio; Herbarium Benesperi | PV187723 | PV423766 |  |
| ecv- 35 | *Cetrelia monachorum* | 10/2022; Toscana: Comano; Herbarium Benesperi | PV187724 | PV423767 | PV423792 |
| ecv- 36 | *Cetrelia monachorum* | 11/2022; Veneto: Cansiglio, Pian Canaje; Herbarium Nascimbene (JN8151) | PV187725 | PV423768 | PV423793 |
| ecv- 37 | *Cetrelia monachorum* | 11/2022; Veneto: Cansiglio, Pian Canaje; Herbarium Nascimbene (JN8152) | PV187726 | PV423769 | PV423794 |
| ecv- 38 | *Cetrelia cetrarioides* | 11/2022; Veneto: Cansiglio, Pian Canaje; Herbarium Nascimbene (JN8155) | PV187727 | PV423770 | PV423795 |
| ecv- 39 | *Cetrelia monachorum* | 11/2022; Veneto: Cansiglio, Campon; Herbarium Nascimbene (JN8148) | PV187728 | PV423771 | PV423796 |
| ecv- 41 | *Cetrelia olivetorum* | 11/2022; Veneto: Cansiglio, Vivaio; Herbarium Nascimbene (JN8132) | PV187729 | PV423772 |  |
| ecv- 42 | *Cetrelia chicitae* | 11/2022; Veneto: Cansiglio, Vivaio; Herbarium Nascimbene (JN8131) | PV187730 | PV423773 | PV423797 |
| ecv- 43 | *Cetrelia monachorum* | 11/2022; Veneto: Longarone, Cajada; Herbarium Nascimbene (JN8117) | PV187731 |  |  |
| ecv- 44 | *Cetrelia olivetorum* | 11/2022; Veneto: Cansiglio, Crosetta; Herbarium Nascimbene (JN8141) | PV187732 | PV423774 | PV423798 |
| ecv- 45 | *Cetrelia monachorum* | 11/2022; Veneto: Cansiglio, Crosetta; Herbarium Nascimbene (JN8142) | PV187733 |  |  |
| ecv- 46 | *Cetrelia monachorum* | 11/2022; Veneto: Cansiglio, Pian Parrocchia; Herbarium Nascimbene (JN8162) | PV187734 | PV423775 | PV423799 |
| ecv- 47 | *Cetrelia monachorum* | 4/2022; Trentino Alto Adige: Tuenno, Sentiero delle Glare; Herbarium Nascimbene (JN8043) | PV187735 |  |  |
| ecv- 48 | *Cetrelia cetrarioides* | 4/2022; Trentino Alto Adige: Tuenno, Sentiero delle Glare; Herbarium Nascimbene (JN8042) | PV187736 | PV423776 | PV423800 |
| ecv- 49 | *Cetrelia chicitae* | 6/2022; Friuli Venezia Giulia: Val Fleons; Herbarium Nascimbene (JN8085) | PV187737 |  |  |
| ecv- 50 | *Cetrelia monachorum* | 5/2022; Trentino Alto Adige: Val Zanca; Herbarium Nascimbene (JN8044) | PV187738 | PV423777 | PV423801 |
| ecv- 51 | *Cetrelia cetrarioides* | 5/2022; Trentino Alto Adige: Val Zanca; Herbarium Nascimbene (JN8047) | PV187739 |  |  |
| ecv- 52 | *Cetrelia monachorum* | 6/2022; Friuli Venezia Giulia: Cima Sappada; Herbarium Nascimbene (JN8075) | PV187740 |  |  |
| ecv-66 | *Cetrelia cetrarioides* | 6/2022; Friuli Venezia Giulia: Sappada; Herbarium Nascimbene (JN8079) | PV187741 |  |  |
| ecv-67 | *Cetrelia monachorum* | 6/2022; Trentino Alto Adige: Tuenno, Lago di Tovel; Herbarium Nascimbene (JN8179) | PV187742 | PV423778 |  |
| ecv-68 | *Cetrelia monachorum* | 6/2022; Friuli Venezia Giulia: Sappada; Herbarium Nascimbene (JN8080) | PV187743 | PV423779 | PV423802 |
| ecv-69 | *Cetrelia cetrarioides* | 6/2022; Trentino Alto Adige: Tuenno, Lago di Tovel; Herbarium Nascimbene (JN8178) | PV187744 | PV423780 |  |
| ecv-70 | *Cetrelia monachorum* | 2/2021; Emilia Romagna: Casa Moschini; Herbarium Benesperi | PV187745 | PV423781 |  |
| ecv-71 | *Cetrelia chicitae* | 8/2023; Lombardia: Schilpario, Pista degli Abeti; Herbarium Gheza | PV187746 | PV423782 | PV423803 |
| ecv-72 | *Cetrelia olivetorum* | 5/2022; Trentino Alto Adige: Val Canali, Acque Negre; Herbarium Nascimbene (JN8050) | PV187747 |  | PV423804 |
