# Supplementary figures and images for: ﻿Towards a better knowledge and conservation of cryptic macrolichens in Italy: a revision of the genus Cetrelia (Parmeliaceae, Lecanorales, lichenized Ascomycota)
Source: MycoKeys. 2025 Aug 8;120:231–54. doi: 10.3897/mycokeys.120.154233 (PMC12357148; doi:10.3897/mycokeys.120.154233)

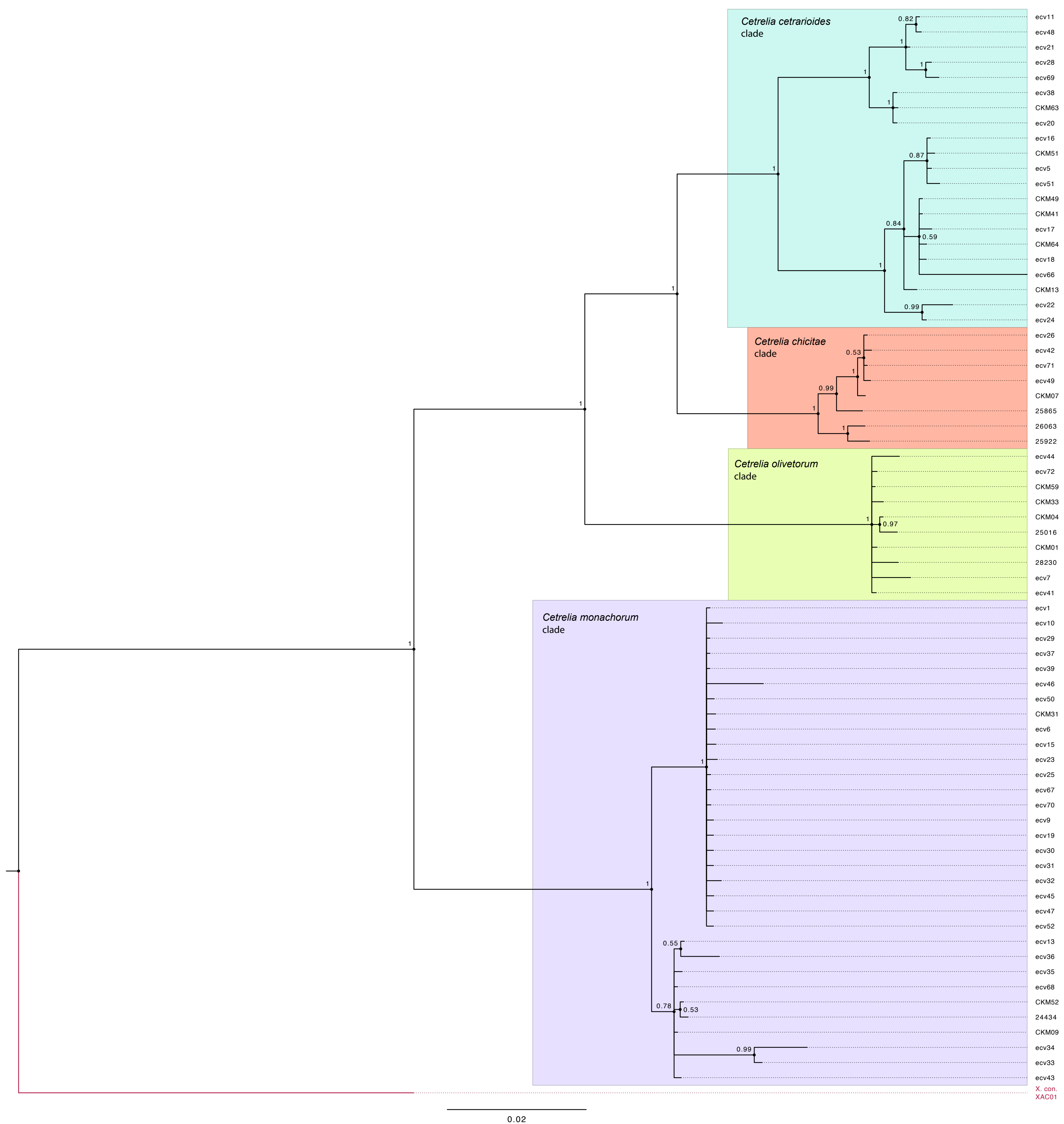

Supplement: Supplementary material 4 — Phylogenetic tree [file mycokeys-120-231-s004.pdf]
