# Supplementary material for: ﻿Towards a better knowledge and conservation of cryptic macrolichens in Italy: a revision of the genus Cetrelia (Parmeliaceae, Lecanorales, lichenized Ascomycota)
Source: MycoKeys. 2025 Aug 8;120:231–54. doi: 10.3897/mycokeys.120.154233 (PMC12357148; doi:10.3897/mycokeys.120.154233)
Supplement: Supplementary material 5 — Macrolichens of conservation interest co-occurring with Cetrelia [file mycokeys-120-231-s005.doc]

**Towards a better knowledge and conservation of cryptic macrolichens in Italy: a revision of the genus *Cetrelia* W.L. Culb. & C.F. Culb.**

Gabriele Gheza, Chiara Vallese, Luca Di Nuzzo, Simona Corneti, Renato Benesperi, Elisabetta Bianchi, Giulia Canali, Silvia Del Vecchio, Luana Francesconi, Paolo Giordani, Pier Luigi Nimis, Walter Obermayer, Chiara Pistocchi, Helmut Mayrhofer, Juri Nascimbene

**Supplementary File 5**

Macrolichens of conservation interest co-occurring with the four *Cetrelia* species in the only three Italian sites hosting all of them. Nomenclature follows Nimis & Martellos (2025).

|  | **Lombardia:**  **Pista degli Abeti** | **Veneto:**  **Foresta del Cansiglio** | **Friuli Venezia Giulia:**  **Lago di Sauris** |
| --- | --- | --- | --- |
| *Alectoria* *sarmentosa* (Ach.) Ach. |  |  | + |
| *Cetraria* *oakesiana* Tuck. |  | + | + |
| *Evernia* *divaricata* (L.) Ach. |  |  | + |
| *Heterodermia* *speciosa* (Wulfen) Trevis. |  | + | + |
| *Hypotrachyna* *laevigata* (Sm.) Hale | + | + | + |
| *Hypotrachyna* *sinuosa* (Sm.) Hale |  |  | + |
| *Leptogium* *cyanescens* (Ach.) Körb. |  | + | + |
| *Lobaria* *pulmonaria* (L.) Hoffm. |  | + | + |
| *Lobarina* *scrobiculata* (Scop.) Cromb. |  | + | + |
| *Menegazzia* *subsimilis* (H. Magn.) R. Sant. |  |  | + |
| *Menegazzia* *terebrata* (Hoffm.) A. Massal. | + | + | + |
| *Nephroma* *bellum* (Spreng.) Tuck. |  | + | + |
| *Nephroma* *laevigatum* Ach. |  | + |  |
| *Nephroma* *parile* (Ach.) Ach. |  | + | + |
| *Nephroma* *resupinatum* (L.) Ach. |  | + | + |
| *Nephromopsis* *laureri* (Kremp.) Kurok. |  | + | + |
| *Pannaria* *conoplea* (Ach.) Bory |  | + | + |
| *Pannaria* *rubiginosa* (Ach.) Bory |  |  | + |
| *Parmelia* *squarrosa* Hale |  |  | + |
| *Parmelia* *submontana* Hale | + | + | + |
| *Parmeliella* *triptophylla* (Ach.) Müll. Arg. |  |  | + |
| *Parmotrema* *arnoldii* (Du Rietz) Hale | + | + | + |
| *Parmotrema* *crinitum* (Ach.) M. Choisy | + | + | + |
| *Peltigera* *collina* (Ach.) Schrad. |  | + | + |
| *Ramalina* *implectens* Nyl. |  |  | + |
| *Ramalina* *obtusata* (Arnold) Bitter |  | + | + |
| *Ramalina* *panizzei* De Not. |  |  | + |
| *Ramalina* *thrausta* (Ach.) Nyl. |  | + | + |
| *Sticta* *fuliginosa* (Hoffm.) Ach. |  |  | + |
| *Sticta* *limbata* (Sm.) Ach. |  | + | + |
| *Sticta* *sylvatica* (Huds.) Ach. |  |  | + |
| *Usnea* *longissima* Ach. |  |  | + |
